# Scoping review of disease-modifying effect of drugs in experimental epilepsy

**DOI:** 10.3389/fneur.2023.1097473

**Published:** 2023-02-23

**Authors:** Heather D. Ots, Taylor Anderson, William Sherrerd-Smith, John DelBianco, Gordana Rasic, Anthony Chuprin, Zeeshan Toor, Elizabeth Fitch, Kripa Ahuja, Faith Reid, Alberto E. Musto

**Affiliations:** ^1^School of Medicine, Eastern Virginia Medical School, Norfolk, VA, United States; ^2^Department of Pathology and Anatomy, Eastern Virginia Medical School, Norfolk, VA, United States; ^3^Department of Neurology, Eastern Virginia Medical School, Norfolk, VA, United States

**Keywords:** seizure, epilepsy, epileptogenesis, anti-seizure medication, neuroprotection, neuroinflammation

## Abstract

**Objective:**

Epilepsy affects ~50 million people worldwide causing significant medical, financial, and sociologic concerns for affected patients and their families. To date, treatment of epilepsy is primarily symptomatic management because few effective preventative or disease-modifying interventions exist. However, recent research has identified neurobiological mechanisms of epileptogenesis, providing new pharmacologic targets to investigate. The current scientific evidence remains scattered across multiple studies using different model and experimental designs. The review compiles different models of anti-epileptogenic investigation and highlights specific compounds with potential epileptogenesis-modifying experimental drugs. It provides a platform for standardization of future epilepsy research to allow a more robust compound analysis of compounds with potential for epilepsy prevention.

**Methods:**

PubMed, Ovid MEDLINE, and Web of Science were searched from 2007 to 2021. Studies with murine models of epileptogenesis and explicitly detailed experimental procedures were included in the scoping review. In total, 51 articles were selected from 14,983 and then grouped by five core variables: (1) seizure frequency, (2) seizure severity, (3) spontaneous recurrent seizures (SRS), (4) seizure duration, and (5) mossy fiber sprouting (MFS). The variables were differentiated based on experimental models including methods of seizure induction, treatment schedule and timeline of data collection. Data was categorized by the five core variables and analyzed by converting original treatment values to units of percent of its respective control.

**Results:**

Discrepancies in current epileptogenesis models significantly complicate inter-study comparison of potential anti-epileptogenic interventions. With our analysis, many compounds showed a potential to reduce epileptogenic characteristics defined by the five core variables. WIN55,212-2, aspirin, rapamycin, 1400W, and LEV + BQ788 were identified compounds with the potential of effective anti-epileptic properties.

**Significance:**

Our review highlights the need for consistent methodology in epilepsy research and provides a novel approach for future research. Inconsistent experimental designs hinder study comparison, slowing the progression of treatments for epilepsy. If the research community can optimize and standardize parameters such as methods of seizure induction, administration schedule, sampling time, and aniMal models, more robust meta-analysis and collaborative research would follow. Additionally, some compounds such as rapamycin, WIN 55,212-2, aspirin, 1400W, and LEV + BQ788 showed anti-epileptogenic modulation across multiple variables. We believe they warrant further study both individually and synergistically.

## Highlights

- Lack of standardization between studies made true comparison difficult, so all compounds were compared as percent difference from the control within its own study.
- This article compares 51 recent studies on experimental pharmacological interventions to prevent epilepsy by comparing their effects on seizure frequency, severity, duration, spontaneous recurrent seizures (SRS), and mossy fiber sprouting (MFS).
- WIN 55,212-2, aspirin, rapamycin, 1400W, and LEV + BQ788 showed promise of effective anti-epileptogenic activity.

## Introduction

Epilepsy, a non-communicable neurological disease characterized by the occurrence of spontaneous recurrent seizures (SRS), affects more than fifty million people worldwide (1). Although generally well-controlled by current antiseizure medications (ASMs), there can be considerable costs, side-effects, and social stigma that accompany the disease.

Although the pathophysiology remains unclear, the changes that turn a normal brain epileptic after a transient insult has been defined as epileptogenesis (2, 3). Over the past decade, investigators have made unprecedented progress in deciphering the intricate cellular and molecular mechanisms of epileptogenesis and epilepsy, targeting therapy based on seizure subtype, and addressing the risks and benefits of mono- and poly-therapy (4, 5). Despite this progress, there is an unmet need of ASMs, more specifically as they relate to treatment resistant epilepsy or prophylactic treatment in individuals with heightened susceptibility to epilepsy (6). Patients with increased susceptibility develop “acquired epilepsy,” which is defined as a symptomatic epilepsy without a genetic or developmental cause (7). An estimated 22.5% of people with epilepsy suffer from pharmacoresistent seizures, which frequently develops after brain trauma (1, 3).

While epileptogenesis has not been fully delineated, it clearly contains ample targets for therapeutic intervention to alter or even prevent its development. However, inconsistent study designs have made it difficult to compare promising interventions that have been discovered. Therefore, to consolidate the most recent data on the topic, this review provides a novel descriptive analysis of published pharmacologic interventions against epileptogenesis. We describe their efficacy as measured by each intervention's effect on seizure frequency, severity, duration, recurrence, and mossy fiber sprouting. These five variables were chosen as they have been identified as hallmarks of epilepsy (see methods) (8–15). Our data showed that, among others, WIN55,212-2, aspirin, rapamycin, 1400W, and LEV + BQ788 show promise for preventative management of epilepsy with potentially synergistic effects.

## Methods

**Figure 1:**
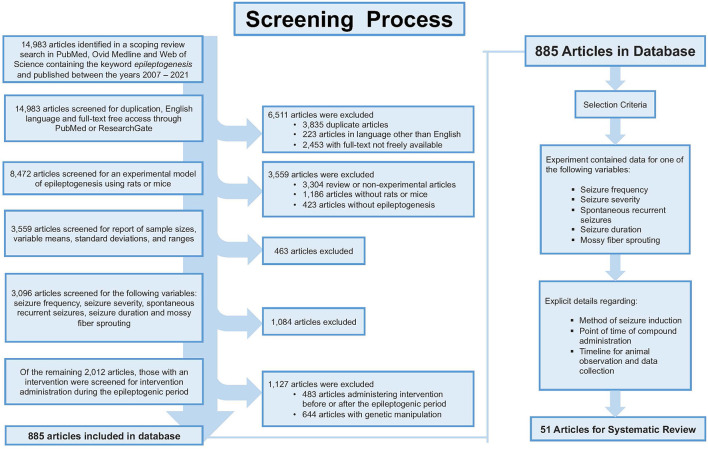
Shows the screening process for article selection. The inclusion and exclusion resulted in 51 articles appropriate for review.

A broad search strategy was developed by a medical research librarian to search three separate databases which included PubMed, Ovid Medline, and Web of Science (Supplementary material). The search was conducted between the years 2007 and 2021, concluding with 14,983 articles total. Articles were selected for review based on strict inclusion and exclusion criteria (Figure 1). The article must have been written in English and freely accessible to the public in full-text format. Treatments must have featured a murine model of epileptogenesis (i.e., using rats or mice). It must have reported on at least one of the following variables: seizure frequency; seizure severity; development of SRS (defined as the number of animals that experienced SRS out of the total number that were subjected to attempted epilepsy induction); seizure duration; or aberrant neuronal plasticity [defined as detected MFS (15, 16)]. Data must have been reported in numbers with sample sizes, means, and standard deviations including time points of data collection and *p*-values. Administration of the intervention must have occurred during the latent period. Due to the variability in study design, we define the latent period as the time after the termination of the status epilepticus (SE) and onset of recurrent seizures (17). Additionally, the article must have provided detailed information regarding the compound, dose, and route and frequency of administration. Using knockout animals or other genetic modifications prior to the onset of epileptogenesis or providing insufficient details of procedures (i.e., experimental intervention or time of data collection, etc.) were criteria for elimination from the study. As a result, a total of 51 articles were selected for final review and analysis.

Data from those articles were grouped by seizure frequency, seizure severity, SRS, seizure duration, and MFS. Seizure frequency is a common variable measured in placebo controlled ASM trials (8). Additionally, seizure severity has been identified as a valuable measurement to assess epilepsy management due to perceived symptomatic improvement (9, 10). The development of SRS was chosen as a measurement to address the progressive nature of acquired epileptogenesis (11, 12). Seizure duration is a contributing factor to epileptogenesis due to its potential to propagate systemic inflammation, leading to dysregulation of the blood-brain-barrier (13). Lastly, due to the cellular pathways that regulate MFS and their potential effects on epileptogenesis, it was selected as a focus variable in our study (14, 15).

**Figure 2:**
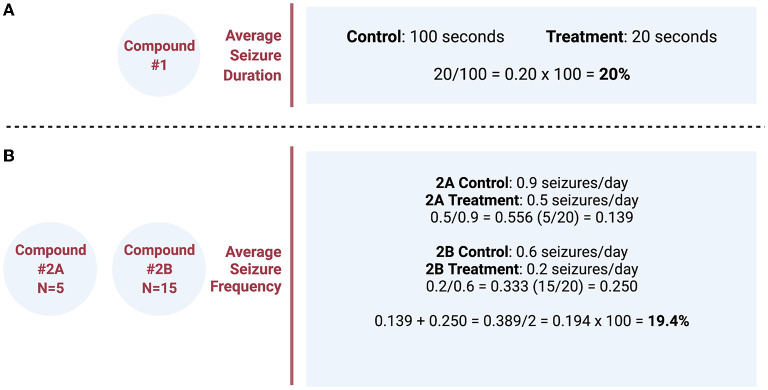
**(A)** Simple example calculation of the quantitative analysis comparing the effect of each pharmacologic compound with respect to its own control. **(B)** Example calculation of two different studies, examining the same compound and dose, that combines the data and weights percentages based on the different sample size (*N*) of each study. These percentages were then plotted for comparison between different pharmacologic interventions grouped by the variable of interest.

True meta-analysis or systematic review could not be performed because the methods of each study varied too greatly. Therefore, to create a different quantitative analysis, the effect of each treatment with respect to its own control was analyzed and plotted for comparison. Only interventions that were identified as statistically significant in the original study were analyzed. Therefore, the source of the *p*-values is from the reported results from the original research. Data analysis compared the percent difference of treatment vs. control using recorded data from each study as described in Figure 2. Briefly, if the average seizure duration was 20 s in the treatment group and 100 s in the control group (vehicle), we reported this as a decrease in seizure duration to 20% of the control group with use of the therapeutic compound (Figure 2A). The combination of data was analyzed if two studies examined the same compound and dose. In these cases, the number of animals in each group (*N*) was divided by the sum of both sample sizes to determine each study's overall percent contribution. The percent contribution was then multiplied by the percent difference of treatment vs. control to ensure that greater weight was given to studies with larger sample sizes (Figure 2B). In some articles for seizure frequency, a conversion was made to seizures per day to facilitate comparison between interventions. The percent difference from each study was then inserted into a table and plotted together in a bar graph displaying the percent change in ascending order.

## Results

**Figure 3:**
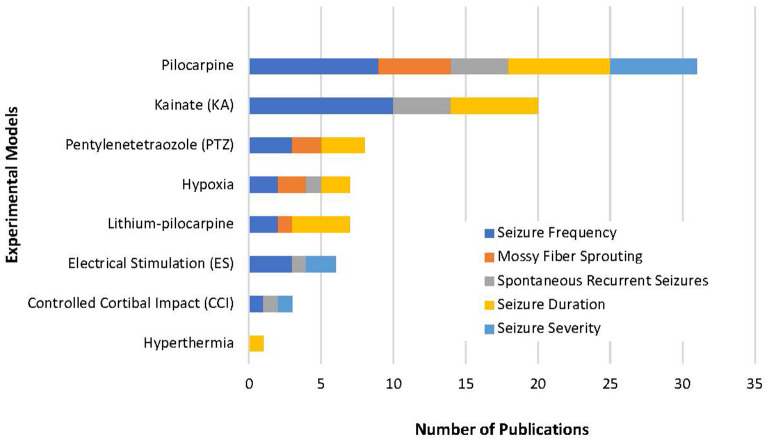
Our review highlights the variety of methods used to study epileptogenesis. This represents the variability of experimental models used in the 51 studies selected and emphasizes how difficult it is to complete a true compound comparison. Of all models, pilocarpine was used in most experimental models and included all five variables in our review. This suggests an experimental model with pilocarpine-induced epilepsy could be used as a standardized approach to the study epileptogenesis.

From the 14,983 articles that resulted from our initial search, a total of 51 articles were eligible for analysis which included a total of 46 compounds (Figure 1). Despite our strict criteria, there was still considerable variability in murine model, methods of seizure induction, timeline of intervention, method of administration and timeline of data collection. Of these articles, 63% used rats and 37% used mice in the experimental protocols. These animal models used induced-SE, kindling, traumatic brain injury (TBI), hypoxia-induced seizures, and febrile seizures models (Figure 3). The time of intervention after the start of the latent period was <24-h in 57%, at 24-h in 27% and >24-h in 16%. Sixty-six percent of compounds were administered *via* intraperitoneal (ip) injection, 10% subcutaneous (sc) injection, 10% intracerebroventricular (icv) injection, 8% oral administration, 4% intragastric administration and 2% intra-amygdala injection. Data was collected both during and after the compound administration in 47%, at the end of compound administration in 49% and simultaneously with the administration of the compound in 4%.

The primary outcomes of our analysis are summarized in Table 1. The data was categorized by the five core variables and compared within each category as follows.

**Table 1:** Displays the percent change from control for each intervention and dose for seizure frequency, severity, spontaneous recurrent seizures (SRS), duration, and mossy fiber sprouting (MFS).

**Drug name**	**Dose**	**Seizure frequency**	**Seizure severity**	**Recurrent seizures**	**Seizure duration**	**Mossy fiber sprouting**
Adenosine	50,000 H239 cells	50.00^**^	X	X	54.00^**^	X
Anakinra	100 mg/kg	X	X	X	26.67^*^	X
Anti-VGKC	0.24 mg/kg	23.33^*^	18.18^*^	X	28.57^*^	X
Aspirin	20 mg/kg	14.29^*^	X	X	28.62^*^	43.59^*^
Biperiden	8 mg/kg	33.33^*^	X	40.27^*^	X	X
Citalopram	15 mg/kg	X	X	68.75^*^	X	X
Citalopram	20 mg/kg	X	X	56.03^*^	X	X
Dapagliflozin	75 mg/kg	X	42.36^**^	X	26.72^**^	X
Dapagliflozin	150 mg/kg	X	38.18^**^	X	17.62^**^	X
FK506	2 mg/kg	76.43^**^	X	X	80.04^**^	X
FGF2 + BDNF	1.6 × 10^6^ pfu	30.77^*^	62.50^*^	X	114.55^*^	X
Glatiramer acetate	150 μg/kg	X	X	42.86^*^	12.73^*^	80.00^*^
GYKI 52466	10 mg/kg	43.69^**^	51.35^**^	X	55.37^**^	X
GYKI 52466	50 mg/kg	X	X	X	11.82^*^	X
HSP990	0.5 mg/kg	7.14^**^	X	X	X	X
JZL184	20 mg/kg	73.08^*^	X	100.00^*^	90.32^*^	X
Lacosamide	10 mg/kg	142.6^*^	X	90.00^*^	111.37^*^	X
Lacosamide	30 mg/kg	127.6^*^	X	90.00^*^	106.39^*^	X
Leptin	4 mg/kg	X	X	X	X	75.00^*^
LEV + BQ788	500 mg/ml + 10 mg/ml	15.22^*^	X	X	0.927^*^	X
LEV + SB202190	500 mg/ml + 0.3 mg/ml	X	X	X	24.33^*^	X
LEV + TPM	200 mg/kg + 30 mg/kg	22.00^**^	X	70.00^*^	X	X
Losartan	10 mg/kg	19.29^*^	X	X	X	X
Lovastatin	20 mg/kg	X	X	X	X	46.56^*^
LSP2-9166	10 mg/kg	41.67^**^	X	X	80.00^*^	X
MDL-28170	50 mg/kg	X	X	50.00^*^	X	X
Melatonin	2.5 mg/kg	50.59^*^	X	X	X	X
Melatonin	8 mg/kg	X	X	X	40.00^**^	X
Melatonin	10 mg/kg	23.96^*^	X	X	X	X
MK-801	0.5 mg/kg	X	X	X	X	54.96^*^
Minocycline	45 mg/kg	X	54.72^*^	X	X	X
Myoinositol	30 mg/kg	43.75^**^	X	X	42.86^**^	X
NBQX	20 mg/kg	4.33^**^	73.68^**^	X	X	79.76^*^
norBNI	5 mg/kg	X	65.00^*^	X	40.00^*^	X
NPD1	570 μg/kg	X	37.97^*^	X	37.90^*^	X
Parecoxib	10 mg/kg	X	86.44^**^	X	X	X
Perampanel	8 mg/kg	53.95^**^	56.76^**^	X	54.02^**^	X
*Pergularia daemia*	12.3 mg/kg	X	X	X	35.46^**^	X
PD1n-3 DPA	200 ng/μl	50.00^*^	X	X	55.00^**^	X
Phenobarbitol	15 mg/kg	13.33^*^	X	X	X	X
Rapamycin	1.5 mg/kg	X	X	X	X	56.52^*^
Rapamycin	3 mg/kg	40.99^*^	X	X	X	43.48^*^
Rapamycin	6 mg/kg	15.00^*^	X	46.00^*^	X	X
Rapamycin	10 mg/kg	X	X	X	X	22.27^*^
Reboxetine	30 mg/kg	61.02^*^	X	X	X	X
Recombinant EPO	5,000 IU/kg/day	X	X	X	53.96^*^	X
Resveratrol	15 mg/kg	X	X	19.07^*^	X	X
RHC80267	1.3 μM	90.00^*^	X	82.51^*^	55.17^*^	X
shGAP43	2 μl	20.00^**^	X	X	X	X
Sodium butyrate	600 mg/kg	X	X	X	X	37.50^*^
TPPU	0.1 mg/kg/day	17.42^**^	X	X	X	X
U50488	5 mg/kg	X	105.00^**^	X	60.00^**^	X
WIN 55,212-2	2 mg/kg	17.61^**^	17.33^**^	X	17.22^**^	X
5-ITU	1.6 mg/kg	61.75^*^	X	X	57.17^*^	X
1400W	20 mg/kg	X	X	7.39^**^	X	X

All values reported as the ratio of the mean experimental value to the mean control value as calculated as: average experimental effect/average control effect.

* Indicates a statistically significant value with a p-value of 0.05 or less.

** Indicates a highly statistically significant value with a p-value of 0.001 or less.

### Seizure frequency

Seizure frequency is highly associated with pharmacological resistance and a decreased quality of life in patients with epilepsy (18). Seizure frequency data was collected from 27 compounds across 30 different studies. One article studied Lacosamide at 10 and 30 mg/kg (19). The average duration of the administration of these compounds was 15 days.

The compounds shown to reduce seizure frequency relative to their respective control group, listed from most effective to least effective, are as follows (Table 2): NBQX (4.33%, *p* = 0.0004) (20), HSP990 (7.14%, *p* < 0.001) (21), phenobarbital (13.33%, *p* < 0.01) (22), aspirin (14.29%, *p* < 0.05) (23), rapamycin at 6 mg/kg (15%, *p* < 0.05) (24–26), levetiracetam (LEV) + BQ788 (15.22%, *p* < 0.05) (27), TPPU (17.42%, *p* = 0.0008) (28), WIN 55,212-2 (17.61%, *p* < 0.0001) (29), losartan (19.29, *p* < 0.05) (30), shGAP43 (20%, *p* = 0.001) (31), LEV + topiramate (TPM; 22%, *p* < 0.01) (32), anti-VGKC (23.33%, *p* < 0.01) (33), melatonin at 10 mg/kg (23.96%, *p* < 0.05) (34, 35), reboxetine (30.5%, *p* < 0.01) (36), fibroblast growth factor 2 (FGF2) + brain-derived neurotrophic factor (BDNF; 30.77%, *p* < 0.01) (37), biperiden (33.33%, *p* = 0.03) (38), rapamycin at 3 mg/kg (40.99%, *p* < 0.05) (39), LSP2-9166 (41.67%, *p* < 0.001) (40), GYKI 52466 at 10 mg/kg (43.69%, *p* = 0.001) (41), myoinositol (43.75%, *p* < 0.001) (42), adenosine (50%, *p* < 0.001) (43), PD1n-3DPA (50%, *p* < 0.05) (44), melatonin at 2.5 mg/kg (50.59%, *p* < 0.05) (45), perampanel (53.95%, *p* = 0.001) (36), 5-iodotubercidin (5-ITU; 61.75%, *p* = 0.04) (46), JZL184 (73.08%, *p* < 0.05) (47), tacrolimus (FK506; 76.43%, *p* < 0.001) (48), and RHC80267 (90%, *p* < 0.05) (47). Interestingly, lacosamide at 30 mg/kg (127.6%, *p* < 0.05) (19) and lacosamide at 10 mg/kg (142.6%, *p* < 0.05) (19) caused an increased seizure frequency compared with control groups.

**Table 2:** Seizure frequency.

**Drug name**	**Dose**	**% from control**	**P-value**
Adenosine	50,000 H239 cells	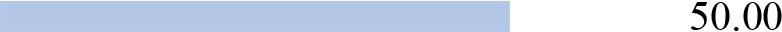	<0.001
Anti-VGKC	0.24 mg/kg	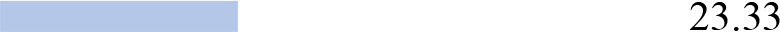	<0.01
Aspirin	20 mg/kg	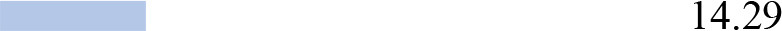	<0.05
Biperiden	8 mg/kg	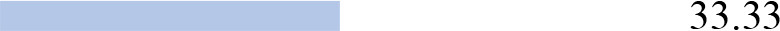	0.03
FK506	2 mg/kg	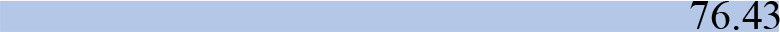	<0.001
FGF2 + BDNF	1.6 × 10^6^ pfu	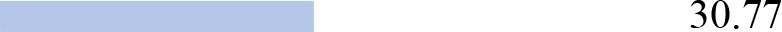	<0.01
GYKI 52466	10 mg/kg	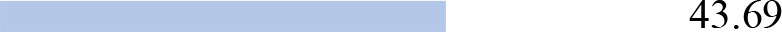	0.001
HSP990	0.5 mg/kg	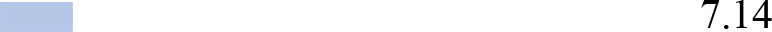	<0.001
JZL184	20 mg/kg	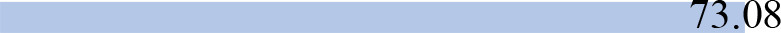	<0.05
Lacosamide	10 mg/kg	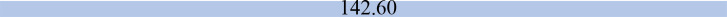	<0.05
Lacosamide	30 mg/kg	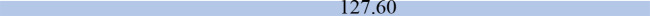	<0.05
LEV + BQ788	500 mg/ml + 10 mg/ml	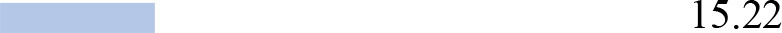	<0.05
LEV + TPM	200 mg/kg + 30 mg/kg	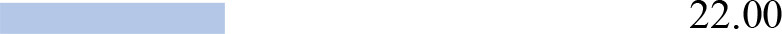	<0.01
Losartan	10 mg/kg	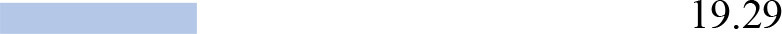	<0.05
LSP2-9166	10 mg/kg	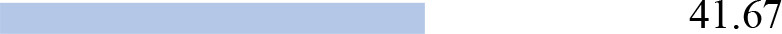	<0.001
Melatonin	2.5 mg/kg	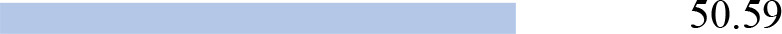	<0.05
Melatonin	10 mg/kg	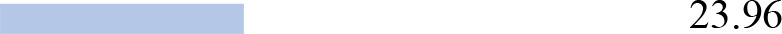	<0.05
Myoinositol	30 mg/kg	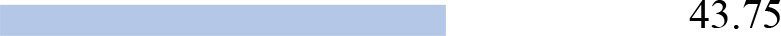	<0.001
NBQX	20 mg/kg	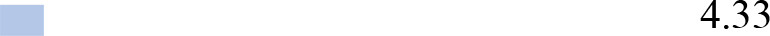	0.0004
Perampanel	8 mg/kg	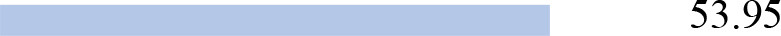	0.001
PD1_n − 3DPA_	200 ng/μl	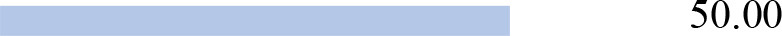	<0.05
Phenobarbital	15 mg/kg	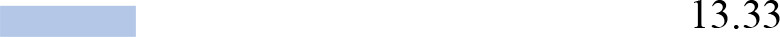	<0.01
Rapamycin	3 mg/kg	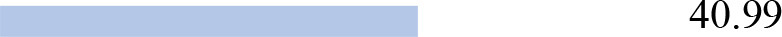	<0.05
Rapamycin	6 mg/kg	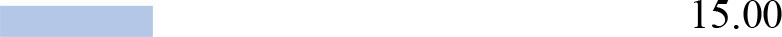	<0.05
Reboxetine	30 mg/kg	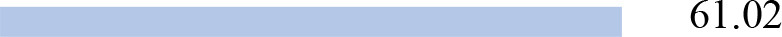	<0.01
RHC80267	1.3 μM	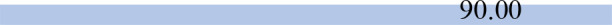	<0.05
shGAP43	2 μl	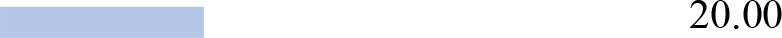	0.001
TPPU	0.1 mg/kg/day	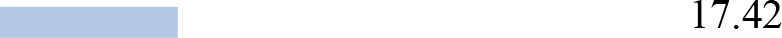	0.0008
WIN55, 212-2	2 mg/kg	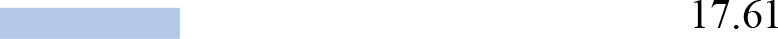	<0.0001
5-ITU	1.6 mg/kg	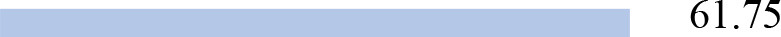	0.04

Percent change from controls for each treatment (horizontal bars) for each intervention and dose as calculated by: (mean experimental value)/(mean control value) and the associated p-value.

### Seizure severity

Seizure severity is positively correlated with the extent of brain damage and comorbidities including psychological, cognitive, and social dysfunction (49). Twelve compounds across 10 different studies were examined for their effect on seizure severity; dapagliflozin was measured at 75 and 150 mg/kg (50). The duration of treatment ranged from 1 to 60 days after compound administration and occurred for an average of 16 days.

The compounds shown to reduce seizure severity relative to their respective control group, reported from most effective to least effective, are as follows (Table 3): WIN 55,212-2 (17.33%, *p* < 0.0001) (29), anti-VGKC (18.18%, *p* < 0.01) (31), neuroprotectin D1 (NPD1; 37.97%, *p* < 0.05) (51), dapagliflozin at 150 mg/kg (38.18%, *p* < 0.001) (50), dapagliflozin at 75 mg/kg (42.36%, *p* < 0.001) (50), GYKI 52466 (51.35%, *p* = 0.001) (41), minocycline (54.72%, *p* < 0.05) (52), perampanel (56.76%, *p* = 0.002) (41), FGF2 + BDNF (62.50%, *p* < 0.05) (37), norBNI (65%, *p* = 0.027) (53), NBQX (73.68%, *p* = 0.001) (54) and parecoxib (86.44%, *p* < 0.001) (55). Finally, U50488, a kappa opioid receptor agonist, was shown to slightly increase seizure severity compared to the control animals (105%, *P* < 0.001) (53).

**Table 3:** Seizure severity.

**Drug name**	**Dose**	**% from control**	**P-value**
Anti-VGKC	0.24 mg/kg	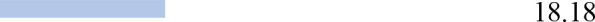	<0.01
Dapagliflozin	75 mg/kg	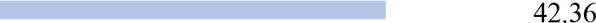	<0.001
Dapagliflozin	150 mg/kg	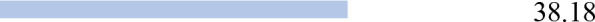	<0.001
FGF2 + BDNF	1.6 × 10^6^ pfu	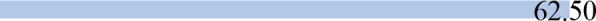	<0.05
GYKI 52466	10 mg/kg	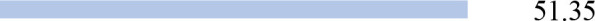	0.001
Minocycline	45 mg/kg	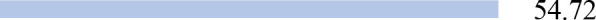	<0.05
NBQX	20 mg/kg	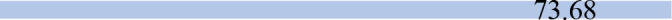	0.001
norBNI	5 mg/kg	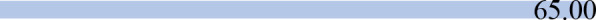	0.027
NPD1	570 μg/kg	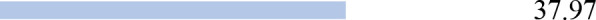	<0.05
Parecoxib	10 mg/kg	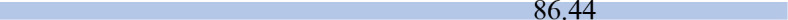	<0.001
Perampanel	8 mg/kg	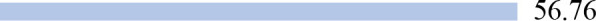	0.002
U50488	5 mg/kg	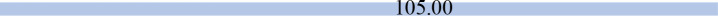	<0.001
WIN 55,212-2	2 mg/kg	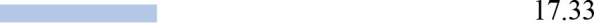	<0.0001

Percent change from controls for each treatment (horizontal bars) for each intervention and dose as calculated by: (mean experimental value)/(mean control value) and the associated p-value.

### Spontaneous recurrent seizures

The development of SRS are a hallmark of epilepsy and a key factor in determining the success of anti-epileptogenic or disease-modifying effects (12). Eleven articles evaluated a total of 11 compounds, some with multiple doses, with respect to SRS. The duration of therapy ranged from 1 to 60 days, with an average of 15 days among all studies examined.

The compounds shown to reduce SRS relative to their respective control group, reported from most effective to least effective, are as follows (Table 4): 1400W (7.39%, *p* < 0.0001) (56), resveratrol (19.07%, *p* < 0.05) (57), biperiden (40.27%, *p* = 0.02) (38), glatiramer acetate (42.86%, *p* < 0.05) (58), rapamycin at 6 mg/kg [46%, *p* < 0.05 (24), *p* = 0.027 (25)], MDL28170 (50%, *p* < 0.05) (59), citalopram at 20 mg/kg (56.03%, *p* < 0.01) (36), citalopram at 15 mg/kg (68.75%, *p* < 0.05) (36), LEV + TPM (70%, *p* ≤ 0.05) (32), RHC80267 (82.51%, *p* < 0.05) (48), lacosamide at 10 mg/kg (90%, *p* < 0.05) (19) and lacosamide at 30 mg/kg (90%, *p* < 0.05) (19). JZL184, an irreversible enzyme inhibitor responsible for degrading 2-arachidonoylglycerol, appeared to produce the equivalent SRS compared to its control (100%, *p* < 0.05) (47).

**Table 4:** Spontaneous recurrent seizures.

**Drug name**	**Dose**	**% from control**	**P-value**
Biperiden	8 mg/kg	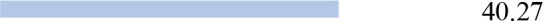	0.02
Citalopram	15 mg/kg	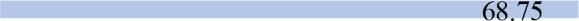	<0.05
Citalopram	20 mg/kg	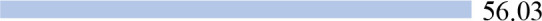	<0.01
Glatiramer Acetate	150 μg/kg	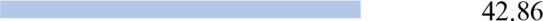	<0.05
JZL184	20 mg/kg	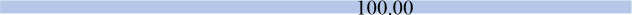	<0.05
Lacosamide	10 mg/kg	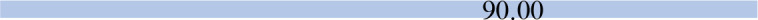	<0.05
Lacosamide	30 mg/kg	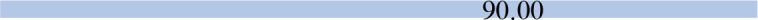	<0.05
LEV + TPM	200 mg/kg + 30 mg/kg	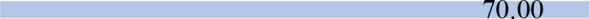	≤0.05
MDL-28170	50 mg/kg	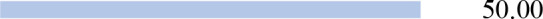	<0.05
Rapamycin	6 mg/kg	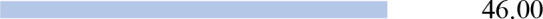	<0.05, 0.027
Resveratrol	15 mg/kg	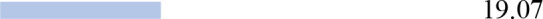	<0.05
RHC80267	1.3 μM	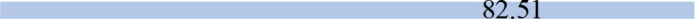	<0.05
1400W	20 mg/kg	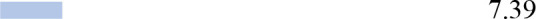	<0.0001

Percent change from controls for each treatment (horizontal bars) for each intervention and dose as calculated by: (mean experimental value)/(mean control value) and the associated p-value.

### Seizure duration

Seizure duration is associated with increased risk of neurotoxicity and cell death (60). Twenty-three articles studying 27 compounds were plotted in our analysis of the effect on seizure duration. On average compounds were administered for 14 days, ranging from 1 to 30 days.

The compounds shown to reduce seizure duration relative to their respective control group, reported from most effective to least effective, are as follows (Table 5): LEV + BQ788 (0.927%, *p* < 0.05) (27), GYKI 52466 at 50 mg/kg (11.82%, *p* < 0.01) (61), glatiramer acetate (12.73%, *p* < 0.01) (58), WIN55,212-2 (17.22%, *p* < 0.0001) (29), dapagliflozin at 150 mg (17.62%, *p* < 0.001) (50), LEV + SB202190 (24.33%, *p* < 0.05) (27), anakinra (26.67%, *p* = 0.031) (62), dapagliflozin at 75 mg/kg (26.72%, *p* < 0.001) (50), anti-VGKC (28.57%, *p* < 0.01) (33), aspirin (28.62%, *p* < 0.01) (23), *Pergularia daemia* (*P*. *daemia*; 35.46%, *p* < 0.01) (63), NPD1 (37.90%, *p* = 0.014) (51), melatonin at 8 mg/kg (40%, *p* < 0.0001) (64), norBNI (40%, *p* < 0.001) (53), myoinositol (42.86%, *p* = 0.001) (42), recombinant erythropoietin (EPO; 53.96%, *p* < 0.05) (65), adenosine (54%, *p* < 0.001) (43), perampanel (54.02%, *p* = 0.001) (41), PD1n-3DPA (55%, *p* < 0.001) (42), RHC80267 (55.17%, *p* < 0.05) (48), GYKI 52466 at 10 mg/kg (55.37%, *p* = 0.002) (41), 5-ITU (57.17%, *p* = 0.0049) (47), U50488 (60%, *p* < 0.001) (53), LSP2-9166 (80%, *p* < 0.05) (40), FK506 (80.04%, *p* < 0.001) (49), and JZL184 (90.32%, *p* < 0.05) (48). The compounds that increased seizure duration were lacosamide at 30 mg/kg (106.39%, *p* < 0.05) (19), lacosamide at 10 mg/kg (111.37%, *p* < 0.05) (19) and FGF2 + BDNF (114.55%, *p* < 0.01) (37).

**Table 5:** Seizure duration.

**Drug name**	**Dose**	**% from control**	**P-value**
Adenosine	50,000 H239 cells	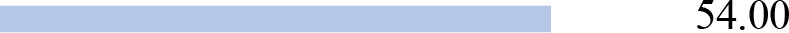	<0.001
Anakinra	100 mg/kg	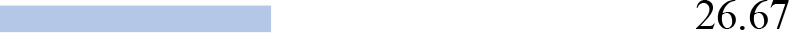	0.031
Anti-VGKC	0.24 mg/kg	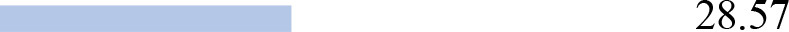	<0.01
Aspirin	20 mg/kg	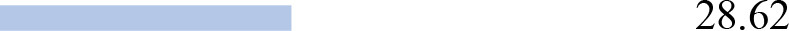	<0.01
Dapagliflozin	75 mg/kg	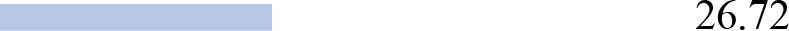	<0.001
Dapagliflozin	150 mg/kg	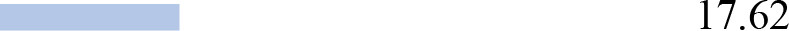	<0.001
FK506	2 mg/kg	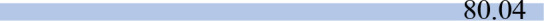	<0.001
FGF2 + BDNF	1.6 × 10^6^ pfu	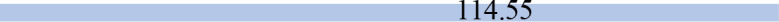	<0.01
Glatiramer Acetate	150 μg/kg	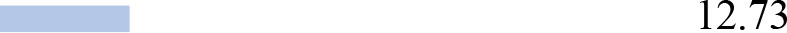	<0.01
GYKI 52466	10 mg/kg	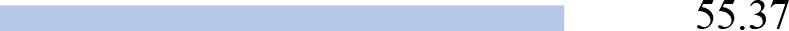	0.002
GYKI 52466	50 mg/kg	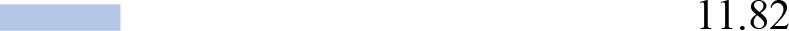	<0.01
JZL184	20 mg/kg	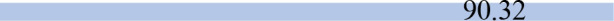	<0.05
Lacosamide	10 mg/kg	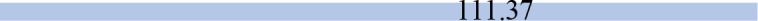	<0.05
Lacosamide	30 mg/kg	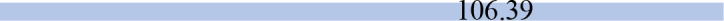	<0.05
LEV + BQ788	500 mg/ml + 10 mg/ml	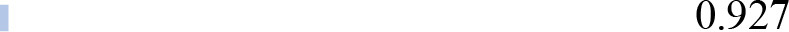	<0.05
LEV + SB202190	500 mg/ml + 0.3 mg/ml	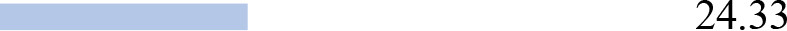	<0.05
LSP2-9166	10 mg/kg	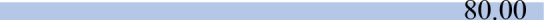	<0.05
Melatonin	8 mg/kg	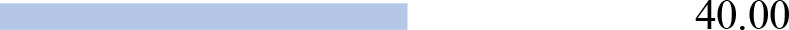	<0.0001
Myoinositol	30 mg/kg	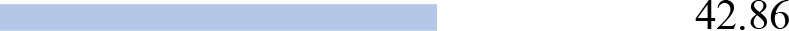	0.001
norBNI	5 mg/kg	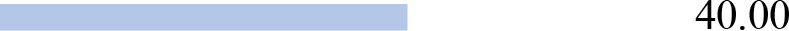	<0.001
NPD1	570 μg/kg	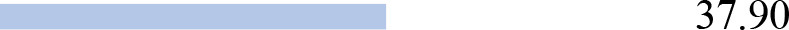	0.014
Perampanel	8 mg/kg	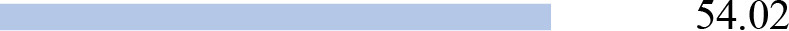	0.001
*P. daemia*	12.3 mg/kg	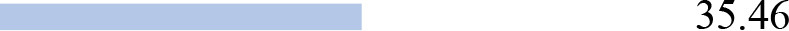	<0.01
PD1n-3 DPA	200 ng/μl	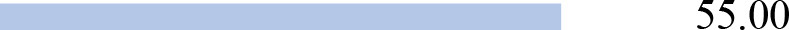	<0.001
Recombinant EPO	5,000 IU/kg/day	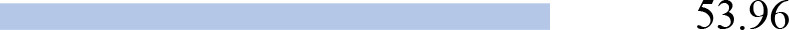	<0.05
RHC80267	1.3 μM	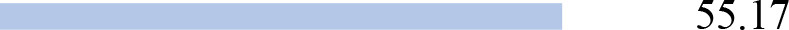	<0.05
U50488	5 mg/kg	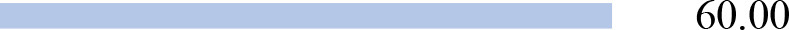	<0.001
WIN 55,212-2	2 mg/kg	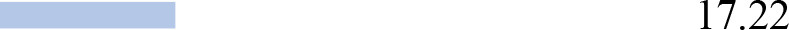	<0.0001
5-ITU	1.6 mg/kg	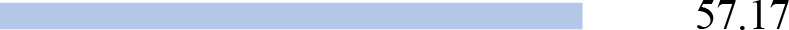	0.0049

Percent change from controls for each treatment (horizontal bars) for each intervention and dose as calculated by: (mean experimental value)/(mean control value) and the associated p-value.

### Mossy fiber sprouting

Mossy fiber growth from the dentate gyrus implies aberrant neuronal plasticity and may serve as a marker of structural alteration of neural circuits that may contribute to epilepsy (15). Nine articles evaluated the effect of eight compounds on the development of these mossy fibers in experimental models of epilepsy. Rapamycin was studied at 1.5, 3 (66), and 10 mg/kg (67, 68). The average duration of treatment was 22 days with ranges from 1 to 56 days of compound administration.

The results were recorded based on the Timm's staining score (TS) from the hippocampal tissue of the treated groups. Timm's stain is a histochemical technique that specifically labels synaptic terminals of mossy fibers through their high zinc content. The extent of mossy fiber sprouting is a measure of aberrant neuronal plasticity and is represented using TS ranging from 1 to 5 according to established criteria (16). The compounds shown to reduce mossy fiber sprouting relative to their respective control group, reported from most effective to least effective, are as follows (Table 6): rapamycin at 10 mg/kg (22.27%, *p* = 0.02, *p* < 0.05) (67, 68), sodium butyrate (37.50%, *p* < 0.05) (69), rapamycin at 3 mg/kg (43.48%, *p* < 0.05) (66), aspirin (43.59%, *p* < 0.01) (23), lovastatin (46.56%, *p* < 0.01) (70), MK801 (54.96%, *p* < 0.01) (70), rapamycin at 1.5 mg/kg (56.52%, *p* < 0.05) (66), leptin (75%, *p* < 0.05) (71), NBQX (79.76%, *p* = 0.036) (20) and glatiramer acetate (80%, *p* < 0.05) (58).

**Table 6:** Mossy fiber sprouting.

**Drug name**	**Dose**	**% from control**	**P-value**
Aspirin	20 mg/kg	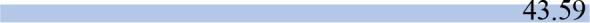	<0.01
Glatiramer Acetate	150 μg/kg	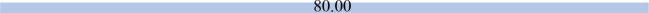	<0.05
Leptin	4 mg/kg	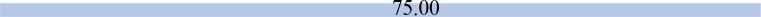	<0.05
Lovastatin	20 mg/kg	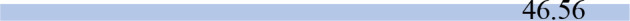	<0.01
MK-801	0.5 mg/kg	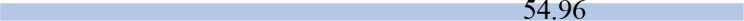	<0.01
NBQX	20 mg/kg	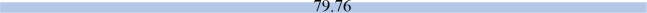	0.036
Rapamycin	1.5 mg/kg	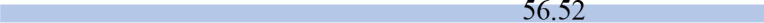	<0.05
Rapamycin	3 mg/kg	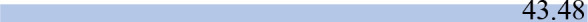	<0.05
Rapamycin	10 mg/kg	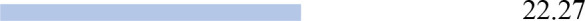	0.02, <0.05
Sodium butyrate	600 mg/kg	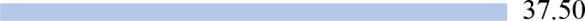	<0.05

Percent change from controls for each treatment (horizontal bars) for each intervention and dose as calculated by: (mean experimental value)/(mean control value) and the associated p-value.

## Discussion

Out of the 51 articles on epileptogenesis selected for analysis, five compounds were highlighted as potential candidates to target the epileptogenic process: LEV + BQ788, WIN55212-2, aspirin, 1400W and rapamycin. This was based on their significant modification (limit or reduction) of the five core variables in this study. Individually, they modulate different pathways leading toward epileptogenesis. Other effective drugs studied in this project showed reductions in isolated variables. For example, recent research has highlighted that adenosine augmentation can significantly reduce mossy fiber sprouting and thus epileptogenesis (72). To help standardize data specific to epileptogenesis, our analysis was limited by excluding models containing knockout animals or genetic modifications that may have highlighted potential antiepileptogenic therapies. For example, genetically altered mice with restrictive deletion of the mechanistic target of rapamycin (mTOR) in microglia (mTOR^Cx3cr1 − cre^CKO), have recently been found to play an important role in the neuroexcitatory pathway of epileptogenesis (73). There have been clinical studies that evaluate the potential antiepileptogenic effects of promising compounds such as phenobarbitol (74). However, the focus of our review is to summarize pre-clinical research and analyze it based on our selection criteria. Notably, of the five selected compounds, the effects of rapamycin were studied at four different doses [1.5 (66), 3 (39, 66), 6 (24–26), and 10 mg/kg (67, 68)] and data was compiled from seven different studies that were independently replicated by four different research groups. Our review was also limited due to focused data from murine models of epilepsy, which did not account for the model- or species-specific findings that may have impacted individual results.

**Figure 4:**
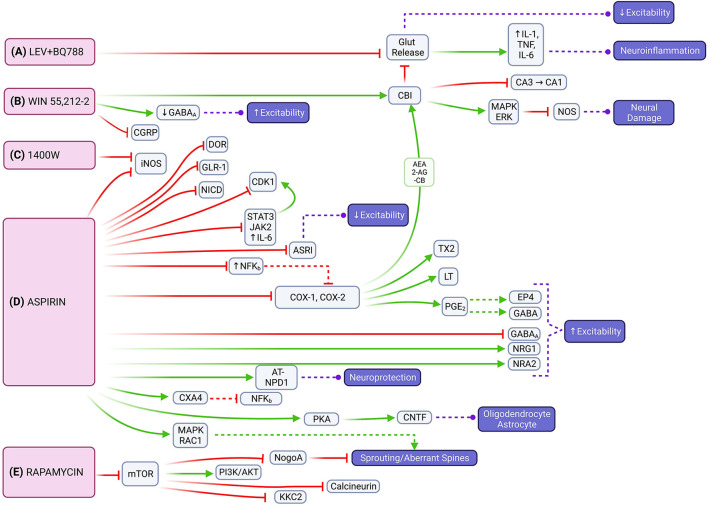
Molecular signaling mediated by LEV + BQ788, WIN 55,212-2, 1400W, aspirin and rapamycin that represents possible targets to mitigate epileptogenesis through multiple pathways. **(A)** The combination of LEV with BQ788 has the potential to attenuate neuroexcitability and neuroinflammation by inhibiting glutamate transmission. **(B)** WIN 55,212-2 acts on the CB1 receptor that leads to the activation of the mitogen-activated protein kinase (MAPK) and retrograde inhibition of neurotransmitter release [e.g., inhibition of nitric oxide synthase (NOS)]. **(C)** 1400W is an inhibitor of iNOS. **(D)** Aspirin also inhibits iNOS, as well as reduces expression of DOR, IL6/JAK2/STAT3, and NFK_B_. Neuroinflammation can be limited through aspirin's inhibition of COX1/COX2 and the endocannabinoid system [e.g., 2-arachidonoylglycerol (2-AG), arachidonoylethanolamine (AEA)]. Neuroprotective effects are instigated through activation of NR2A or PKA with subsequent CNTF release. **(E)** Through its effects on mTOR signaling, rapamycin has the potential to regulate axonal fiber regeneration *via* NogoA. It also reduces expression of NMDA and AMPA receptor subunits and down-regulates expression of PSD-95 and KCC2.

Our review revealed the combination of LEV with BQ788, an endothelin receptor antagonist, reduces seizure frequency and duration in chronic epileptic rats that showed no response to LEV alone (27). Despite its anti-inflammatory properties, LEV has not yet proven itself to prevent epilepsy (75, 76). However, by blocking glutamate release and attenuating the inflammatory response, there is potential to reduce neuroexcitability and neuroinflammation (Figure 4A). Interestingly, a recent clinical trial showed LEV has potential protective properties against posttraumatic epilepsy in the pediatric population (77). Discrepancies between results may be due to pre-clinical vs. clinical application.

WIN 55,212-2 had the most consistent decrease across seizure frequency, seizure severity, and seizure duration based on the analyzed studies. It works as a cannabinoid agonist that acts mainly at the CB1 receptor expressed highly in the hippocampus and cerebellum (78). CB1 receptor activation also reduces spontaneous firing of hippocampal neurons in glutamatergic, GABAergic, and cholinergic signaling (79). Most cannabinoids primarily increase glutamate levels in the prefrontal cortex, dorsal striatum, nucleus accumbens, and hippocampus; WIN 55,212-2 additionally decreases glutamate levels in the amygdala and hypothalamus (80). Through downstream activation of CB1 receptors or attenuating hyperexcitability by suppressing glutamate levels, WIN 55,212-2 has the potential to impair epileptogenesis by modulating both excitatory and inflammatory pathways that contribute to seizure susceptibility (Figure 4B). Cannabidiols have shown promise as ASMs in recent clinical trials (81). Most recently, the University of Colorado has a clinical trial related to the use of medicinal cannabinoids as an alternative treatment option for refractory epilepsy (82).

Chronic glial activation by proinflammatory cytokines and reactive oxygen/nitrogen species (ROS/RNS) can lead to a cycle of neurodegeneration and hyperexcitability, resulting in a lower threshold for seizures (83, 84) (Figure 4C). 1400W, a highly selective pharmacological inhibitor of inducible nitric oxide synthase (iNOS), was found to significantly reduce the number of SRS and attenuate blood-brain-barrier (BBB) impairment (56). By attenuating the inflammatory response caused by glial cells in the early post-SE period, the anti-neuroinflammatory and neuroprotectant effects of 1400W have the potential to alter the disease progression in acquired epilepsy (85). Clinical trials with 1400W are related to its cardiovascular effects, not its relationship to seizures or the nervous system.

Aspirin is a non-selective cyclooxygenase (COX) inhibitor that impairs the metabolic pathway converting arachidonic acid (AA) to prostaglandins, which are important mediators of neuroinflammation. Physiologic upregulation of this inflammatory pathway increases neuronal excitability and decreased seizure threshold, which suggests that limiting neuroinflammation could slow epileptogenesis (Figure 4D). Our review showed that aspirin was able to decrease seizure frequency, seizure duration, and aberrant neuronal plasticity. Aspirin, similar to 1400W, has also been found to inhibit iNOS (86). It decreases delta-opioid receptor (DOR) expression in the cortex, but not in the hippocampus, which contributes to synaptic homeostasis and may limit seziure propagation in epileptogenesis (53). Aspirin also modulates the expression of inflammatory pathways through the downregulation of nuclear factor kappa B (NF-KB), interleukin 6 (IL-6), janus kinase 2 (JAK2), signal transducer and activator of transcription 2 (STAT3) (87). Aspirin limits neuroinflammation *via* the COX1 and COX2 pathways as well as reduces prostaglandin E2 (PGE_2_) which allows gamma-aminobutyric acid (GABA) signaling to evoke robust neuronal activity under inflammatory conditions (88). Additionally, aspirin's metabolite, salicylate, reduces neuronal inhibition through GABA-A and glycine receptors and inhibits high-conductance calcium-activated and voltage-dependent potassium channels (89, 90). Of note, inhibition of COX-2 increases endocannabinoid tone and facilitates endocannabinoid-dependent inhibition of synaptic transmission, which limits neuroinflammation indirectly by reducing glutamate release (91, 92). This allows for a beneficial interaction between aspirin and WIN55,212-2 for impairing epileptogenesis. Aspirin also increases *N*-methyl-D-aspartate receptor (NMDAR) subunit 2A (NR2A) expression in the hippocampus and enhances currents by increasing AA levels (93). It also acts as a neuroprotective factor *via* aspirin-triggered neuroprotectin D1 (AT-NPD1) and astroglial ciliary neurotrophic factor (CNTF) (94, 95). Although the excitatory verse inhibitory drive is unbalanced, excitatory effects of aspirin treatment could compensate for the lack of neuronal activity that arises because of SE- induced neuron damage. Aspirin and salicylate may mitigate neuronal damage by inhibition of acid-sensing ion channels that would otherwise be activated in acidosis-induced injury (96). Prior studies examining COX inhibitors found varying degrees of success in limiting neuroinflammation in SE (97). The conflicting results regarding aspirin's effect on neuronal excitability may be due to variance in experimental conditions. However, it is possible that aspirin may cause hyperexcitability itself in certain circumstances if there is an imbalance in its excitatory and inhibitory effects, and the balance is tilted toward excitation. Further studies are needed to clarify the effect of aspirin in epileptogenesis. Clinical trials of aspirin's anti-epileptogenic effects are currently focused on patients with refractory seizures in the context of tuberous sclerosis complex (TSC) (98).

Rapamycin, an allosteric mTOR inhibitor, showed its ability to decrease seizure frequency, development of SRS, and aberrant MFS. Though some experiments with rapamycin have found low statistical significance (66, 67), our overall analysis showed rapamycin to have a relatively consistent benefit across multiple doses and variables. The mTOR pathway is important in epilepsy pathophysiology (Figure 4E). Hyperactivation of mTOR is correlated with a high occurrence of epileptic seizures and the promotion of activity-dependent mRNA translation near synapses that play a critical role in neuronal circuit formation (26). NogoA, a myelin-associated protein, is associated with axonal fiber regeneration following injury and regulated through the mTOR signaling pathway (99). One of two heteromeric mTOR complexes, mTORC2, regulates the actin cytoskeleton and F-actin nerve fibers (100). Modification of these nerve fibers and dendritic spines (DS) is associated with cognitive impairment in temporal lobe epilepsy (TLE). DS could be the first subcellular site of aberrant neuronal networks in limbic epileptogenesis (LE) because they represent the morphological signature of postsynaptic sites and excitatory synaptic transmission and the sub-cellular neural component for neuronal network assemblies (101). Overexpression of the *N*-methyl-D-aspartate (NMDA) receptor in DS could trigger neuronal hyper-excitability and induce LE (102). Rapamycin reduces expression of NMDA and AMPA receptor subunits and dendritic postsynaptic density protein 95 (PSD-95) and decreases synapse density in the dentate gyrus following epileptogenesis (26, 103). This functionality suggests the role of aberrant post-synaptic modification in the development of epilepsy and a potential anti-epileptogenic mechanism for rapamycin. Additionally, rapamycin modulates neuronal excitability by down-regulating the expression of potassium-chloride cotransporter 2 (KCC2) in the thalamic-hippocampal network (104). Rapamycin offers added neuroprotective benefit as systemic administration protects perforant pathway projections from tau-mediated neurodegeneration, axonal and synapse loss, and neuroinflammatory gliosis (105). Similar to aspirin, clinical trials studying rapamycin and epilepsy are focused on its use in pharmacoresistent epilepsy associated with TSC (106, 107).

These compounds participate in different cellular and molecular mechanisms that are postulated in the pathogenesis of epilepsy (108). However, our results allow us to examine the molecular signaling pathways that have the potential for anti-epileptogenic effects as we demonstrate in Figure 4.

Overall, our review highlights the need for a standardized methodological approach to epileptogenesis research and variability between research articles makes compound analysis/comparison, and essentially true meta-analysis, impossible. For example, some of the early rapamycin work was experimentally flawed, because seizure recordings were done during the presence of rapamycin without considering a drug-washout periods, which is needed to validate antiepileptogenic effects (109). If researchers adopted techniques and examined variables that are consistent with other experiments, it would allow for better comparison between studies and expansion upon prior results. As a starting point, we recommend that compound administration occur during the latent period following the SE or initial seizure induction because this most accurately parallels clinical use. Following any given event known to increase seizure susceptibility, patients could be administered one of the compounds studied for its antiepileptogenic mechanism to decrease the likelihood of downstream epilepsy development. Compound administration during the latent period was used as an inclusion criterion for this paper and more reliable evidence and more compounds could have been compared if more studies used this timeline. Additionally, for consistency, we propose studying these compounds on rats and mice, using one as the standard model of epilepsy and the other to validate interventions. Finally, we recommend timing drug-administration appropriately so that data recording can begin immediately after administration. Without a standardized approach to administration and data collection, there is not a methodologically or statistically sound way to compare any experimental compound against another, and it is difficult to make applications and inferences as to future clinical implementation and effect. Ideally more thought will go into comparison of data collection schedules and methodology, and a discussion will arise to determine how best to study epileptogenesis. Without these changes, collaboration, and comparison of data on this subject will continue to hinder the pursuit of treatments.

## Author contributions

AM conceived the idea, figures, tables and search, and wrote and review the manuscript. HO, TA, WS-S, JD, GR, AC, ZT, EF, KA, and FR conducted the search, analysis, wrote the manuscript, and contribute with figures and tables. HO and TA equally contributed to the review of the literature and writing of the manuscript. All authors contributed to the article and approved the submitted version.

## References

[1] Kwan P Schachter SC Brodie MJ. Drug-resistant epilepsy. N Engl J Med. (2011) 365:919–26. 10.1056/NEJMra1004418 21899452

[2] Pitkänen A Lukasiuk K Dudek FE Staley KJ. Epileptogenesis. Cold Spring Harb Perspect Med. (2015) 5:a022822. 10.1101/cshperspect.a022822 26385090 PMC4588129

[3] Becker AJ. Review: animal models of acquired epilepsy: insights into mechanisms of human epileptogenesis. Neuropathol Appl Neurobiol. (2018) 44:112–9. 10.1111/nan.12451 29130506

[4] Vezzani A Balosso S Ravizza T. Neuroinflammatory pathways as treatment targets and biomarkers in epilepsy. Nat Rev Neurol. (2019) 15:459–72. 10.1038/s41582-019-0217-x 31263255

[5] Manford M. Recent advances in epilepsy. J Neurol. (2017) 264:1811–24. 10.1007/s00415-017-8394-2 28120042 PMC5533817

[6] Chen Z Brodie MJ Liew D Kwan P. Treatment outcomes in patients with newly diagnosed epilepsy treated with established and new antiepileptic drugs: a 30-year longitudinal cohort study. JAMA Neurol. (2018) 75:279–86. 10.1001/jamaneurol.2017.3949 29279892 PMC5885858

[7] Shorvon SD. The etiologic classification of epilepsy. Epilepsia. (2011) 52:1052–7. 10.1111/j.1528-1167.2011.03041.x 21449936

[8] Clinical investigation of medicinal products in the treatment of epileptic disorders. Eur Neuropsychopharmacol. (2001) 11:253–9. 10.1016/S0924-977X(01)00085-2 11418286

[9] Aghaei-Lasboo A Fisher RS. Methods for measuring seizure frequency and severity. Neurol Clin. (2016) 34:383–94, viii. 10.1016/j.ncl.2015.11.001 27086985

[10] Smith D Baker G Davies G Dewey M Chadwick DW. Outcomes of add-on treatment with lamotrigine in partial epilepsy. Epilepsia. (1993) 34:312–22. 10.1111/j.1528-1157.1993.tb02417.x 8453943

[11] Williams PA White AM Clark S Ferraro DJ Swiercz W Staley KJ. Development of spontaneous recurrent seizures after kainate-induced status epilepticus. J Neurosci. (2009) 29:2103–12. 10.1523/JNEUROSCI.0980-08.2009 19228963 PMC2897752

[12] Gu B Dalton KA. Models and detection of spontaneous recurrent seizures in laboratory rodents. Zool Res. (2017) 38:171–9. 10.24272/j.issn.2095-8137.2017.042 28825447 PMC5571473

[13] Fauser S Tumani H. Epilepsy. Handb Clin Neurol. (2018) 14:259–66. 10.1016/B978-0-12-804279-3.00015-0 29110774

[14] Godale CM Danzer SC. Signaling pathways and cellular mechanisms regulating mossy fiber sprouting in the development of epilepsy. Front Neurol. (2018) 9:298. 10.3389/fneur.2018.00298 29774009 PMC5943493

[15] Sutula T. Seizure-induced axonal sprouting: assessing connections between injury, local circuits, and epileptogenesis. Epilepsy Curr. (2002) 2:86–91. 10.1111/j.1535-7597.2002.00032.x 15309153 PMC321023

[16] Cavazos JE Cross DJ. The role of synaptic reorganization in mesial temporal lobe epilepsy. Epilepsy Behav. (2006) 8:483–93. 10.1016/j.yebeh.2006.01.011 16500154 PMC2829602

[17] Lévesque M Biagini G de Curtis M Gnatkovsky V Pitsch J Wang S. The pilocarpine model of mesial temporal lobe epilepsy: over one decade later, with more rodent species and new investigative approaches. Neurosci Biobehav Rev. (2021) 130:274–91. 10.1016/j.neubiorev.2021.08.020 34437936

[18] Birbeck GL Hays RD Cui X Vickrey BG. Seizure reduction and quality of life improvements in people with epilepsy. Epilepsia. (2002) 43:535–8. 10.1046/j.1528-1157.2002.32201.x 12027916

[19] Licko T Seeger N Zellinger C Russmann V Matagne A Potschka H. Lacosamide treatment following status epilepticus attenuates neuronal cell loss and alterations in hippocampal neurogenesis in a rat electrical status epilepticus model. Epilepsia. (2013) 54:1176–85. 10.1111/epi.12196 23614482

[20] Lippman-Bell JJ Rakhade SN Klein PM Obeid M Jackson MC Joseph A. AMPA receptor antagonist NBQX attenuates later-life epileptic seizures and autistic-like social deficits following neonatal seizures. Epilepsia. (2013) 54:1922–32. 10.1111/epi.12378 24117347 PMC4262152

[21] Sha L Chen T Deng Y Du T Ma K Zhu W. Hsp90 inhibitor HSP990 in very low dose upregulates EAAT2 and exerts potent antiepileptic activity. Theranostics. (2020) 10:8415–29. 10.7150/thno.44721 32724478 PMC7381737

[22] Brandt C Nozadze M Heuchert N Rattka M Löscher W. Disease-modifying effects of phenobarbital and the NKCC1 inhibitor bumetanide in the pilocarpine model of temporal lobe epilepsy. J Neurosci. (2010) 30:8602–12. 10.1523/JNEUROSCI.0633-10.2010 20573906 PMC6634618

[23] Ma L Cui X-L Wang Y Li X-W Yang F Wei D. Aspirin attenuates spontaneous recurrent seizures and inhibits hippocampal neuronal loss, mossy fiber sprouting and aberrant neurogenesis following pilocarpine-induced status epilepticus in rats. Brain Res. (2012) 1469:103–13. 10.1016/j.brainres.2012.05.058 22765917

[24] van Vliet EA Forte G Holtman L den Burger JCG Sinjewel A de Vries HE. Inhibition of mammalian target of Rapamycin reduces epileptogenesis and blood-brain barrier leakage but not microglia activation. Epilepsia. (2012) 53:1254–63. 10.1111/j.1528-1167.2012.03513.x 22612226

[25] Guo D Zeng L Brody DL Wong M. Rapamycin attenuates the development of posttraumatic epilepsy in a mouse model of traumatic brain injury. PLoS ONE. (2013) 8:e64078. 10.1371/journal.pone.0064078 23691153 PMC3653881

[26] Zeng LH Rensing NR Wong M. The mammalian target of Rapamycin signaling pathway mediates epileptogenesis in a model of temporal lobe epilepsy. J Neurosci. (2009) 29:6964–72. 10.1523/JNEUROSCI.0066-09.2009 19474323 PMC2727061

[27] Ko AR Kang TC. Blockade of endothelin B receptor improves the efficacy of levetiracetam in chronic epileptic rats. Seizure. (2015) 31:133–40. 10.1016/j.seizure.2015.07.019 26362390

[28] Shen Y Peng W Chen Q Hammock BD Liu J Li D. Anti-inflammatory treatment with a soluble epoxide hydrolase inhibitor attenuates seizures and epilepsy-associated depression in the LiCl-pilocarpine post-status epilepticus rat model. Brain Behav Immun. (2019) 81:535–44. 10.1016/j.bbi.2019.07.014 31306773 PMC6873816

[29] Di Maio R Cannon JR Greenamyre JT. Post-status epilepticus treatment with the cannabinoid agonist WIN 55,212-2 prevents chronic epileptic hippocampal damage in rats. Neurobiol Dis. (2015) 73:356–65. 10.1016/j.nbd.2014.10.018 25447228

[30] Tchekalarova JD Ivanova NM Pechlivanova DM Atanasova D Lazarov N Kortenska L. Antiepileptogenic and neuroprotective effects of Losartan in kainate model of temporal lobe epilepsy. Pharmacol Biochem Behav. (2014) 127:27–36. 10.1016/j.pbb.2014.10.005 25456349

[31] Nemes AD Ayasoufi K Ying Z Zhou QG Suh H Najm IM. Growth associated protein 43 (GAP-43) as a novel target for the diagnosis, treatment and prevention of epileptogenesis. Sci Rep. (2017) 7:17702. 10.1038/s41598-017-17377-z 29255203 PMC5735087

[32] Schidlitzki A Bascuñana P Srivastava PK Welzel L Twele F Töllner K. Proof-of-concept that network pharmacology is effective to modify development of acquired temporal lobe epilepsy. Neurobiol Dis. (2020) 134:104664. 10.1016/j.nbd.2019.104664 31678583

[33] Fan Z Feng X Fan Z Zhu X Yin S. Immunotherapy by targeting of VGKC complex for seizure control and prevention of cognitive impairment in a mouse model of epilepsy. Mol Med Rep. (2018) 18:169–78. 10.3892/mmr.2018.9004 29749462 PMC6059666

[34] Tchekalarova J Petkova Z Pechlivanova D Moyanova S Kortenska L Mitreva R. Prophylactic treatment with melatonin after status epilepticus: effects on epileptogenesis, neuronal damage, and behavioral changes in a kainate model of temporal lobe epilepsy. Epilepsy Behav. (2013) 27:174–87. 10.1016/j.yebeh.2013.01.009 23435277

[35] Petkova Z Tchekalarova J Pechlivanova D Moyanova S Kortenska L Mitreva R. Treatment with melatonin after status epilepticus attenuates seizure activity and neuronal damage but does not prevent the disturbance in diurnal rhythms and behavioral alterations in spontaneously hypertensive rats in kainate model of temporal lobe epilepsy. Epilepsy Behav. (2014) 31:198–208. 10.1016/j.yebeh.2013.12.013 24440891

[36] Vermoesen K Massie A Smolders I Clinckers R. The antidepressants citalopram and reboxetine reduce seizure frequency in rats with chronic epilepsy. Epilepsia. (2012) 53:870–8. 10.1111/j.1528-1167.2012.03436.x 22429158

[37] Bovolenta R Zucchini S Paradiso B Rodi D Merigo F Mora GN. Hippocampal FGF-2 and BDNF overexpression attenuates epileptogenesis-associated neuroinflammation and reduces spontaneous recurrent seizures. J Neuroinflammation. (2010) 7:81. 10.1186/1742-2094-7-81 21087489 PMC2993685

[38] Bittencourt S Ferrazoli E Valente MF Romariz S Janisset NRLL Macedo CE. Modification of the natural progression of epileptogenesis by means of biperiden in the pilocarpine model of epilepsy. Epilepsy Res. (2017) 138:88–97. 10.1016/j.eplepsyres.2017.10.019 29096134

[39] Drion CM Borm LE Kooijman L Aronica E Wadman WJ Hartog AF. Effects of rapamycin and curcumin treatment on the development of epilepsy after electrically induced status epilepticus in rats. Epilepsia. (2016) 57:688–97. 10.1111/epi.13345 26924447

[40] Girard B Tuduri P Moreno MP Sakkaki S Barboux C Bouschet T. The mGlu7 receptor provides protective effects against epileptogenesis and epileptic seizures. Neurobiol Dis. (2019) 129:13–28. 10.1016/j.nbd.2019.04.016 31051234

[41] Kim JE Lee DS Park H Kim TH Kang TC. Inhibition of AKT/GSK3β/CREB pathway improves the responsiveness to AMPA receptor antagonists by regulating GRIA1 Surface expression in chronic epilepsy rats. Biomedicines. (2021) 9:425. 10.3390/biomedicines9040425 33919872 PMC8103519

[42] Tsverava L Kandashvili M Margvelani G Lortkipanidze T Gamkrelidze G Lepsveridze E. Long-term effects of myoinositol on behavioural seizures and biochemical changes evoked by kainic acid induced epileptogenesis. Biomed Res Int. (2019) 2019:4518160. 10.1155/2019/4518160 30941363 PMC6421025

[43] Li T Ren G Kaplan DL Boison D. Human mesenchymal stem cell grafts engineered to release adenosine reduce chronic seizures in a mouse model of CA3-selective epileptogenesis. Epilepsy Res. (2009) 84:238–41. 10.1016/j.eplepsyres.2009.01.002 19217263 PMC2746090

[44] Frigerio F Pasqualini G Craparotta I Marchini S van Vliet EA Foerch P. n-3 Docosapentaenoic acid-derived protectin D1 promotes resolution of neuroinflammation and arrests epileptogenesis. Brain. (2018) 141:3130–43. 10.1093/brain/awy247 30307467 PMC6202571

[45] Lima E Cabral FR Cavalheiro EA Naffah-Mazzacoratti M Amado D. Melatonin administration after pilocarpine-induced status epilepticus: a new way to prevent or attenuate postlesion epilepsy? Epilepsy Behavior. (2011) 20:607–12. 10.1016/j.yebeh.2011.01.018 21454134

[46] Sandau US Yahya M Bigej R Friedman JL Saleumvong B Boison D. Transient use of a systemic adenosine kinase inhibitor attenuates epilepsy development in mice. Epilepsia. (2019) 60:615–25. 10.1111/epi.14674 30815855 PMC6713278

[47] Ma L Wang L Yang F Meng XD Wu C Ma H. Disease-modifying effects of RHC80267 and JZL184 in a pilocarpine mouse model of temporal lobe epilepsy. CNS Neurosci Therap. (2014) 20:905–15. 10.1111/cns.12302 24989980 PMC6493031

[48] Xiong TQ Chen LM Tan BH Guo CY Li YN Zhang YF. The effects of calcineurin inhibitor FK506 on actin cytoskeleton, neuronal survival and glial reactions after pilocarpine-induced status epilepticus in mice. Epilepsy Res. (2018) 140:138–47. 10.1016/j.eplepsyres.2018.01.007 29358156

[49] Harden CL Maroof DA Nikolov B Fowler K Sperling M Liporace J. The effect of seizure severity on quality of life in epilepsy. Epilepsy Behav. (2007) 11:208–11. 10.1016/j.yebeh.2007.05.002 17604229

[50] Erdogan MA Yusuf D Christy J Solmaz V Erdogan A Taskiran E. Highly selective SGLT2 inhibitor dapagliflozin reduces seizure activity in pentylenetetrazol-induced murine model of epilepsy. BMC Neurol. (2018) 18:81. 10.1186/s12883-018-1086-4 29879920 PMC5991447

[51] Musto AE Walker CP Petasis NA Bazan NG. Hippocampal neuro-networks and dendritic spine perturbations in epileptogenesis are attenuated by neuroprotectin d1. PLoS ONE. (2015) 10:e0116543. 10.1371/journal.pone.0116543 25617763 PMC4305283

[52] Wang N Mi X Gao B Gu J Wang W Zhang Y. Minocycline inhibits brain inflammation and attenuates spontaneous recurrent seizures following pilocarpine-induced status epilepticus. Neuroscience. (2015) 287:144–56. 10.1016/j.neuroscience.2014.12.021 25541249

[53] Queenan BN Dunn RL Santos VR Feng Y Huizenga MN Hammack RJ. Kappa opioid receptors regulate hippocampal synaptic homeostasis and epileptogenesis. Epilepsia. (2018) 59:106–22. 10.1111/epi.13941 29114861 PMC5774867

[54] Rakhade SN Zhou C Aujla PK Fishman R Sucher NJ Jensen FE. Early alterations of AMPA receptors mediate synaptic potentiation induced by neonatal seizures. J Neurosci. (2008) 28:7979–90. 10.1523/JNEUROSCI.1734-08.2008 18685023 PMC2679369

[55] Polascheck N Bankstahl M Löscher W. The COX-2 inhibitor parecoxib is neuroprotective but not antiepileptogenic in the pilocarpine model of temporal lobe epilepsy. Exp Neurol. (2010) 224:219–33. 10.1016/j.expneurol.2010.03.014 20353773

[56] Puttachary S Sharma S Verma S Yang Y Putra M Thippeswamy A. 1400W, a highly selective inducible nitric oxide synthase inhibitor is a potential disease modifier in the rat kainate model of temporal lobe epilepsy. Neurobiol Dis. (2016) 93:184–200. 10.1016/j.nbd.2016.05.013 27208748

[57] Wu Z Xu Q Zhang L Kong D Ma R Wang L. Protective effect of resveratrol against kainate-induced temporal lobe epilepsy in rats. Neurochem Res. (2009) 34:1393–400. 10.1007/s11064-009-9920-0 19219549

[58] Lai MC Lin KM Yeh PS Wu SN Huang CW. The novel effect of immunomodulator-glatiramer acetate on epileptogenesis and epileptic seizures. Cell Physiol Biochem. (2018) 50:150–68. 10.1159/000493965 30278465

[59] Lam PM Carlsen J González MI. A calpain inhibitor ameliorates seizure burden in an experimental model of temporal lobe epilepsy. Neurobiol Dis. (2017) 102:1–10. 10.1016/j.nbd.2017.02.003 28237317 PMC5640433

[60] Naylor DE. Treating acute seizures with benzodiazepines: does seizure duration matter? Epileptic Disord. (2014) 16 Spec No 1:S69–83. 10.1684/epd.2014.0691 25323468

[61] Fritsch B Stott JJ Joelle Donofrio J Rogawski MA. Treatment of early and late kainic acid-induced status epilepticus with the noncompetitive AMPA receptor antagonist GYKI 52466. Epilepsia. (2010) 51:108–17. 10.1111/j.1528-1167.2009.02205.x 19682025 PMC4535693

[62] Dyomina AV Zubareva OE Smolensky IV Vasilev DS Zakharova MV Kovalenko AA. Anakinra reduces epileptogenesis, provides neuroprotection, and attenuates behavioral impairments in rats in the lithium-pilocarpine model of epilepsy. Pharmaceuticals. (2020) 13:340. 10.3390/ph13110340 33113868 PMC7692198

[63] Kavaye Kandeda A Okomolo Moto FC Omam Omam JP Mbomo Ayissi RE Ojong L Ngo Bum E. *Pergularia daemia* alters epileptogenesis and attenuates cognitive impairment in kainate-treated mice: insight into anti-inflammatory mechanisms. Epilepsy Behav. (2021) 115:107707. 10.1016/j.yebeh.2020.107707 33429138

[64] Ma Y Sun X Li J Jia R Yuan F Wei D. Melatonin alleviates the epilepsy-associated impairments in hippocampal LTP and spatial learning through rescue of surface GluR2 expression at hippocampal CA1 synapses. Neurochem Res. (2017) 42:1438–48. 10.1007/s11064-017-2200-5 28214985

[65] Jung K-H Chu K Lee S-T Park K-I Kim J-H Kang K-M. Molecular alterations underlying epileptogenesis after prolonged febrile seizure and modification by erythropoietin. Epilepsia. (2011) 52:541–50. 10.1111/j.1528-1167.2010.02916.x 21269282

[66] Buckmaster PS Lew FH. Rapamycin suppresses mossy fiber sprouting but not seizure frequency in a mouse model of temporal lobe epilepsy. J Neurosci. (2011) 31:2337–47. 10.1523/JNEUROSCI.4852-10.2011 21307269 PMC3073836

[67] Butler CR Boychuk JA Smith BN. Effects of rapamycin treatment on neurogenesis and synaptic reorganization in the dentate gyrus after controlled cortical impact injury in mice. Front Syst Neurosci. (2015) 9:163. 10.3389/fnsys.2015.00163 26640431 PMC4661228

[68] Heng K Haney MM Buckmaster PS. High-dose Rapamycin blocks mossy fiber sprouting but not seizures in a mouse model of temporal lobe epilepsy. Epilepsia. (2013) 54:1535–41. 10.1111/epi.12246 23848506 PMC3769425

[69] Reddy SD Clossen BL Reddy DS. Epigenetic histone deacetylation inhibition prevents the development and persistence of temporal lobe epilepsy. J Pharmacol Exp Ther. (2018) 364:97–109. 10.1124/jpet.117.244939 29101217

[70] Lee C-Y Jaw T Tseng H-C Chen I-C Liou H-H. Lovastatin modulates glycogen synthase kinase-3β pathway and inhibits mossy fiber sprouting after pilocarpine-induced status epilepticus. PLoS ONE. (2012) 7:e38789. 10.1371/journal.pone.0038789 22761705 PMC3383707

[71] Ni H Chen SH Li LL Jin MF. Leptin treatment prevents long-term abnormalities in cognition, seizure threshold, hippocampal mossy fiber sprouting and ZnT3/CB-D28k expression in a rat developmental “twist” seizure model. Epilepsy Res. (2018) 139:164–70. 10.1016/j.eplepsyres.2017.12.009 29287786

[72] Williams-Karnesky RL Sandau US Lusardi TA Lytle NK Farrell JM Pritchard EM. Epileptogenetic changes induced by adenosine augmentation therapy prevent epileptogenesis. J Clin Invest. (2013) 123:3552–63. 10.1172/JCI65636 23863710 PMC3726154

[73] Zhao XF Liao Y Alam MM Mathur R Feustel P Mazurkiewicz JE. Microglial mTOR is neuronal protective and antiepileptogenic in the pilocarpine model of temporal lobe epilepsy. J Neurosci. (2020) 40:7593–608. 10.1523/JNEUROSCI.2754-19.2020 32868461 PMC7531547

[74] Practice parameter: antiepileptic drug treatment of posttraumatic seizures. Brain Injury Special Interest Group of the American Academy of Physical Medicine and Rehabilitation. Arch Phys Med Rehabil. (1998) 79:594–7. 10.1016/S0003-9993(98)90081-8 9596407

[75] Lee DS Ryu HJ Kim JE Choi HC Kim YI Song HK. The effect of levetiracetam on status epilepticus-induced neuronal death in the rat hippocampus. Seizure. (2013) 22:368–77. 10.1016/j.seizure.2013.02.005 23490457

[76] Bauer J Ben-Menachem E Krämer G Fryze W Da Silva S Kasteleijn-Nolst Trenité DG. Levetiracetam: a long-term follow-up study of efficacy and safety. Acta Neurol Scand. (2006) 114:169–76. 10.1111/j.1600-0404.2006.00657.x 16911344

[77] Pearl PL McCarter R McGavin CL Yu Y Sandoval F Trzcinski S. Results of phase II levetiracetam trial following acute head injury in children at risk for posttraumatic epilepsy. Epilepsia. (2013) 54:e135–7. 10.1111/epi.12326 23876024 PMC3769484

[78] Lauckner JE Hille B Mackie K. The cannabinoid agonist WIN55,212-2 increases intracellular calcium *via* CB1 receptor coupling to Gq/11 G proteins. Proc Natl Acad Sci U S A. (2005) 102:19144–9. 10.1073/pnas.0509588102 16365309 PMC1323208

[79] Deshpane LS Blair RE DeLorenzo RJ. Prolonged cannabinoid exposure alters GABA(A) receptor mediated synaptic function in cultured hippocampal neurons. Exp Neurol. (2011) 229:264–73. 10.1016/j.expneurol.2011.02.007 21324315 PMC3100418

[80] Can Den Pol AN Obrietan K Beousov A. Glutamate hyperexcitability and seizure-like activity throughout the brain and spinal cord upon relief from chronic glutamate receptor blockade in culture. Neuroscience. (1996) 74:653–74. 10.1016/0306-4522(96)00153-4 8884763

[81] Silvestro S Mammana S Cavalli E Bramanti P Mazzon E. Use of cannabidiol in the treatment of epilepsy: efficacy and security in clinical trials. Molecules. (2019) 24:1459. 10.3390/molecules24081459 31013866 PMC6514832

[82] National Library of Medicine (U.S.). The Use of Medicinal Cannabinoids as Adjunctive Treatment for Medically Refractory Epilepsy. Identifier: NCT02523183. Available online at: https://clinicaltrials.gov/ct2/show/NCT02523183 (accessed December 18, 2022).

[83] Papageorgiou IE Fetani AF Lewen A Heinemann U Kann O. Widespread activation of microglial cells in the hippocampus of chronic epileptic rats correlates only partially with neurodegeneration. Brain Struct Funct. (2015) 220:2423–39. 10.1007/s00429-014-0802-0 24878824

[84] Waldbaum S Patel M. Mitochondria, oxidative stress, and temporal lobe epilepsy. Epilepsy Res. (2010) 88:23–45. 10.1016/j.eplepsyres.2009.09.020 19850449 PMC3236664

[85] Loane DJ Kumar A Stoica BA Cabatbat R Faden AI. Progressive neurodegeneration after experimental brain trauma: association with chronic microglial activation. J Neuropathol Exp Neurol. (2014) 73:14–29. 10.1097/NEN.0000000000000021 24335533 PMC4267248

[86] Berk M Dean O Drexhage H McNeil JJ Moylan S O'Neil A. Aspirin: a review of its neurobiological properties and therapeutic potential for mental illness. BMC Med. (2013) 11:74. 10.1186/1741-7015-11-74 23506529 PMC3751197

[87] Li W-Y Li F-M Zhou Y-F Wen Z-M Ma J Ya K. Aspirin down regulates hepcidin by inhibiting NF-κB and IL6/KAJ2/STAT3 pathways in BV-2 microglial cells treated with lipopolysaccharide. Int J Mol Sci. (2016) 17:1921. 10.3390/ijms17121921 27999284 PMC5187761

[88] Jang IJ Davies AJ Akimoto N Back SK Lee PR Na HS. Acute inflammation reveals GABAA receptor-mediated nociception in mouse dorsal root ganglion neurons *via* PGE_2_ receptor 4 signaling. Physiol Rep. (2017) 5:e13178. 10.14814/phy2.13178 28438981 PMC5408276

[89] Lu J Lobarinas E Deng A Goodey R Stolzberg D Salvi RJ. GABAergic neural activity involved in salicylate-induced auditory cortex gain enhancement. Neuroscience. (2011) 189:187–98. 10.1016/j.neuroscience.2011.04.073 21664433 PMC3153886

[90] Liu Y-H Zhang Z-P Wang Y Song J Ma K-T Si J-Q. Electrophysiological properties of strial pericytes and the effect of aspirin on pericyte K+ channels. Mol Med Rep. (2018) 17:2861–8. 10.3892/mmr.2017.8194 29257229 PMC5783500

[91] Wang J Shen RY Haj-Dahmane S. Endocannabinoids mediate the glucocorticoid-induced inhibition of excitatory synaptic transmission to dorsal raphe serotonin neurons. J Physiol. (2012) 590:5795–808. 10.1113/jphysiol.2012.238659 22946098 PMC3528992

[92] Marchalant Y Rosi S Wenk GL. Anti-inflammatory property of the cannabinoid agonist WIN-55212-2 in a rodent model of chronic brain inflammation. Neuroscience. (2007) 144:1516–22. 10.1016/j.neuroscience.2006.11.016 17178196 PMC1852513

[93] Senol N Ceyhan BM Ersoy IH Senol A Acarturk G Sutcu R. Aspirin increases NMDA receptor subunit 2A concentrations in rat hippocampus. J Recept Signal Transduct Res. (2012) 32:17–21. 10.3109/10799893.2011.641975 22171557

[94] Michel BE Kaufmann MR. The osmotic potential of polyethylene glycol 6000. Plant Physiol. (1973) 51:914–6. 10.1104/pp.51.5.914 16658439 PMC366375

[95] Bazan NG Eady TN Khoutorova L Atkins KD Hong S Lu Y. Novel aspirin-triggered neuroprotectin D1 attenuates cerebral ischemic injury after experimental stroke. Exp Neurol. (2012) 236:122–30. 10.1016/j.expneurol.2012.04.007 22542947 PMC3409566

[96] Riley DC Bittner GD Mikesh M Cardwell NL Pollins AC Ghergherehchi CL. Polyethylene glycol-fused allografts produce rapid behavioral recovery after ablation of sciatic nerve segments. J Neurosci Res. (2015) 93:572–83. 10.1002/jnr.23514 25425242 PMC4329031

[97] Holtman L van Vliet EA van Schaik R Queiroz CM Aronica E Gorter JA. Effects of SC58236, a selective COX-2 inhibitor, on epileptogenesis in a rat model for temporal lobe epilepsy. Epilepsy Res. (2009) 84:56–66. 10.1016/j.eplepsyres.2008.12.006 19186029

[98] National Library of Medicine (U.S.). A Placebo-controlled Study of Efficacy and Safety of Aspirin as an add-on Treatment in Patients With Tuberous Sclerosis Complex (TSC) and Refractory Seizures. Identifier: NCT03356769. Available online at a: https://clinicaltrials.gov/ct2/show/NCT03356769 (accessed December 18, 2022).

[99] Zhong J Li X Wan L Chen Z Zhong S Xiao S. Knockdown of NogoA prevents MPP+-induced neurotoxicity in PC12 cells *via* the mTOR/STAT3 signaling pathway. Mol Med Rep. (2016) 13:1427–33. 10.3892/mmr.2015.4637 26648565

[100] Jacinto E Loewith R Schmidt A Lin S Rüegg MA Hall A. Mammalian TOR complex 2 controls the actin cytoskeleton and is Rapamycin insensitive. Nat Cell Biol. (2004) 6:1122–8. 10.1038/ncb1183 15467718

[101] Yuste R Urban R. Dendritic spines and linear networks. J Physiol Paris. (2004) 98:479–86. 10.1016/j.jphysparis.2005.09.014 16309899

[102] Colciaghi F Finardi A Nobili P Locatelli D Spigolon G Battaglia GS. Progressive brain damage, synaptic reorganization and NMDA activation in a model of epileptogenic cortical dysplasia. PLoS ONE. (2014) 9:e89898. 10.1371/journal.pone.0089898 24587109 PMC3937400

[103] Peng X Kim J Zhou Z Fink DJ Mata M. Neuronal Nog-A regulates glutamate receptor subunit expression in hippocampal neurons. J Neurochem. (2011) 119:1183–93. 10.1111/j.1471-4159.2011.07520.x 21985178 PMC3235679

[104] Huang X McMahon J Yang J Shin D Huang Y. Rapamycin down-regulates KCC2 expression and increases seizure susceptibility to convulsants in immature rats. Neuroscience. (2012) 219:33–47. 10.1016/j.neuroscience.2012.05.003 22613737 PMC3402618

[105] Liu S Shen Y Shultz SR Nguyen A Hovens C Adlard PA. Accelerated kindling epileptogenesis in Tg4510 tau transgenic mice, but not in tau knockout mice. Epilepsia. (2017) 58:e136–41. 10.1111/epi.13847 28710841

[106] National Library of Medicine (U.S.). A Placebo-controlled Study of Efficacy and Safety of Rapamycin in Drug Resistant Epilepsy Associated With Tuberous Sclerosis Complex (TSC) and Refractory Seizures. Identifier: NCT05534672. Available online at: https://clinicaltrials.gov/ct2/show/NCT05534672 (accessed December 18, 2022).

[107] National Library of Medicine (U.S.). Efficacy and Safety of Rapamycin Versus Vigabatrin in the Prevention of Tuberous Sclerosis Complex Symptoms in Infants. Identifier: NCT04987463. Available online at: https://clinicaltrials.gov/ct2/show/NCT04987463 (accessed December 18, 2022).

[108] Devinsky O Vezzani A O'Brien TJ Jette N Scheffer IE de Curtis M. Epilepsy. Nat Rev Dis Primers. (2018) 4:18024. 10.1038/nrdp.2018.24 29722352

[109] Gericke B Brandt C Theilmann W Welzel L Schidlitzki A Twele F. Selective inhibition of mTORC1/2 or PI3K/mTORC1/2 signaling does not prevent or modify epilepsy in the intrahippocampal kainate mouse model. Neuropharmacology. (2020) 162:107817. 10.1016/j.neuropharm.2019.107817 31654704

