# Supplementary material for: Scoping review of disease-modifying effect of drugs in experimental epilepsy
Source: Front Neurol. 2023 Feb 23;14:1097473. doi: 10.3389/fneur.2023.1097473 (PMC9997527; doi:10.3389/fneur.2023.1097473)
Supplement: Supplementary file 1 [file Data_Sheet_1.docx]

**Scoping review of disease-modifying effect of drugs in experimental epilepsy**

**PubMed:**

(((("therapeutics"[MH] OR "drug therapy"[MH] OR "drug therapy, combination"[MH] OR therapy[TIAB] OR therapeutic[TIAB] OR therapeutics[TIAB] OR intervention[TIAB] OR interventions[TIAB] OR "drug therapy"[TIAB] OR "drug treatment"[TIAB] OR "drug treatments"[TIAB] OR "drug therapies"[TIAB] OR "mossy fibers, hippocampal"[MH] OR "mossy fiber"[TW] OR "mossy fiber"[TIAB] OR "mossy fibers"[TIAB] OR "mossy fibers"[TW] OR "mossy fibre"[TW] OR "mossy fibre"[TIAB] OR "mossy fiber sprouting"[TIAB] OR "mossy fiber sprouting"[TW]) AND ("rats"[MH] OR "mice"[MH] OR "murinae"[MH] OR rat[TIAB] OR rat[TW] OR rats[TIAB] OR rats[TW] OR mice[TIAB] OR mice[TW] OR mouse[TIAB] OR mouse[TW] OR "mouse model"[TIAB] OR "mouse model"[TW] OR "mouse models"[TIAB] OR "mouse models"[TW] OR "mice model"[TIAB] OR "mice model"[TW] OR "mice models"[TIAB] OR "mice models"[TW] OR mus[TIAB] OR mus[TW] OR murine[TIAB] OR murine[TW] OR "rat models"[TIAB] OR "rat models"[TW] OR "rat model"[TIAB] OR "rat model"[TW] OR "laboratory rat"[TIAB] OR "laboratory rat"[TW] OR "laboratory rats"[TIAB] OR "laboratory rats"[TW] OR "laboratory mice"[TIAB] OR "laboratory mice"[TW])) AND ("seizures"[MH] OR "seizures, febrile"[MH] OR seizure[TIAB] OR seizures[TIAB])) AND ("status epilepticus"[MH] OR "epilepsy"[MH] OR "epilepsy, temporal lobe"[MH] OR "epigenesis, genetic"[MH] OR "status epilepticus"[TIAB] OR epilepsy[TIAB] OR "genetic epigenesis"[TIAB] OR epileptogenesis[TIAB] OR epileptogenic[TIAB] OR epigenesis[TIAB] OR epileptic[TIAB])) AND (("2007"[Date - Publication] : "2021"[Date - Publication])) = 2825 results

**Ovid Medline:**

Ovid MEDLINE(R) <1946 to April Week 5 2021>

1 exp status epilepticus/ or exp epilepsy/ or exp epilepsy, temporal lobe/ or exp epigenesis, genetic/ 254894

2 (status epilepticus or epilepsy or genetic epigenesis or epileptogenesis or epileptogenic or epigenesis or epileptic).ti,ab,kw. 122114

3 1 or 2 285099

4 exp seizures/ or exp seizures, febrile/ 65508

5 (seizure or seizures).ti,ab,kw. 110713

6 4 or 5 134884

7 exp rats/ or exp mice/ or exp murinae/ 3088604

8 (rat or rats or mice or mouse or mouse model or mouse models or mice model or mice models or mus or murine or murinae or rat model or rat models or laboratory rat or laboratory rats or laboratory mice).ti,ab,kw,tw. 2516720

9 7 or 8 3258612

10 exp therapeutics/ or exp drug therapy/ or exp drug therapy, combination/ 4717595

11 (therapy or therapeutic or therapeutics or intervention or interventions or drug therapy or drug treatment or drug treatments or drug therapies).ti,ab,kw. 3074181

12 exp mossy fibers, hippocampal/ 1181

13 (mossy fibre or mossy fiber or mossy fibers or mossy fibre or mossy fibres or (mossy adj fiber adj sprouting)).ti,ab,kw,tw. 4127

14 10 or 11 or 12 or 13 6581616

15 3 and 6 and 9 and 14 4315

16 limit 15 to yr="2007 -Current" 2487

**Web of Science:**


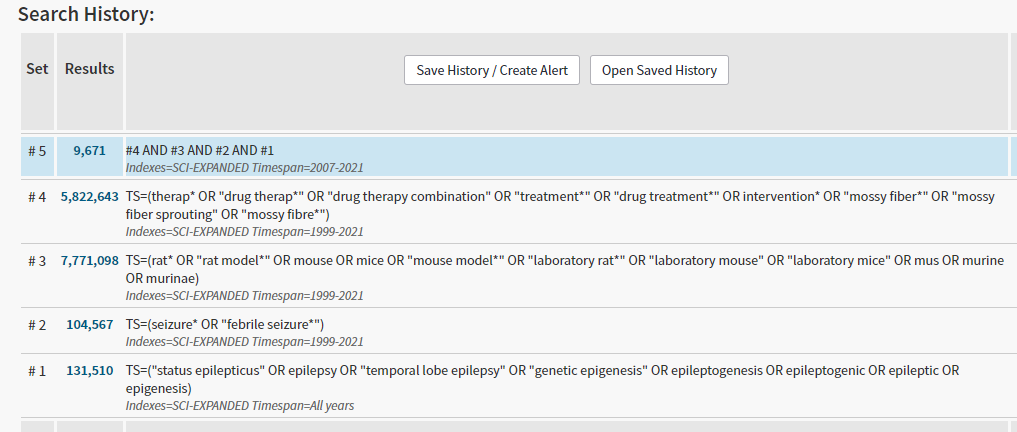


Total of 14983 citations amongst the three of the above databases were collected
